# Supplementary material for: Structure of human spermine oxidase in complex with a highly selective allosteric inhibitor
Source: Commun Biol. 2022 Aug 5;5:787. doi: 10.1038/s42003-022-03735-9 (PMC9355956; doi:10.1038/s42003-022-03735-9)
Supplement: Supplementary file 2 — Supplementary Information [file 42003_2022_3735_MOESM2_ESM.pdf]

Supplementary information:

Structure of human Spermine Oxidase in complex with a highly selective allosteric inhibitor

Elsie Diaz[1*], Suraj Adhikary[1*], Armand W.J.W. Tepper[2*], Daniel Riley[1], Rodrigo Ortiz-Meoz[1], Daniel Krosky[1], Christophe Buyck[3], Carolina Martinez Lamenca[3], Josep Llaveria[4], Lichao Fang[5], Jay H. Kalin[1], Vincent N.A. Klaren[2], Shorouk Fahmy[2], Paul L. Shaffer[1], Robert Kirkpatrick[1], Rodrigo J. Carbajo[4], Maren Thomsen[6] and Antonietta Impagliazzo[2#†]

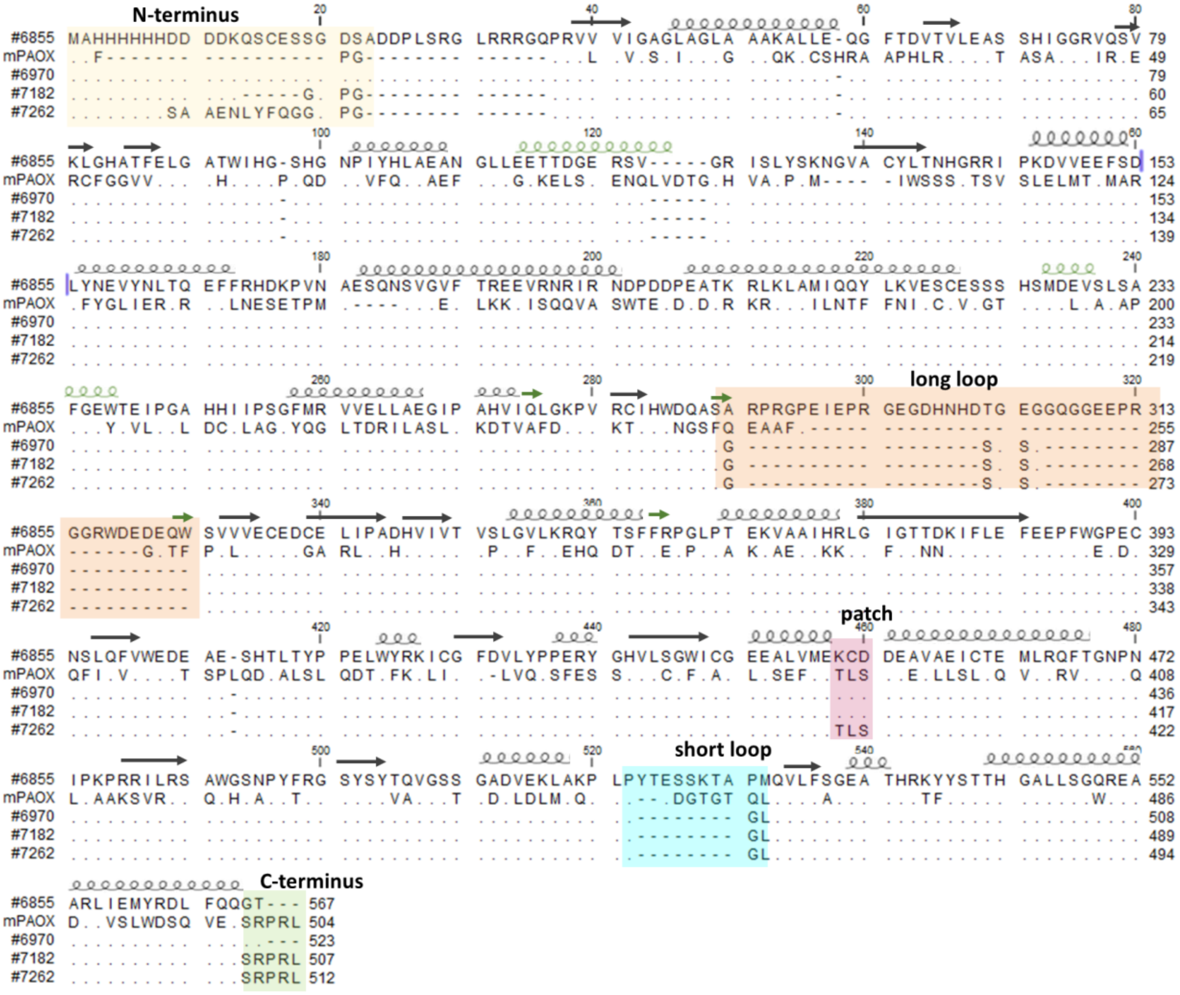

Supplementary Fig.1. Sequence alignment of the main hSMOX engineered constructs. His-tag wt hSMOX (#6855; ID: Q9NWM0-1 UniProtKB) and mPAOX sequence derived from mPAOX structure (PDB ID: 5LAE) are aligned with engineered sequence #6970 and #7182 of stages I and II respectively Fig. 2c. Construct #7262 (Fig. 2c stage III) corresponds to the hSMOX engendered constructs crystallized. Dots indicate identical residues, while dashes indicate missing residue in the sequence. Structural elements are indicated above the sequence in black for both hSMOX and mPAOX. The additional structural elements present in mPAOX are depicted in green.

Colored boxes include part of the sequences that have been engineered using the color code of Figure 2c.

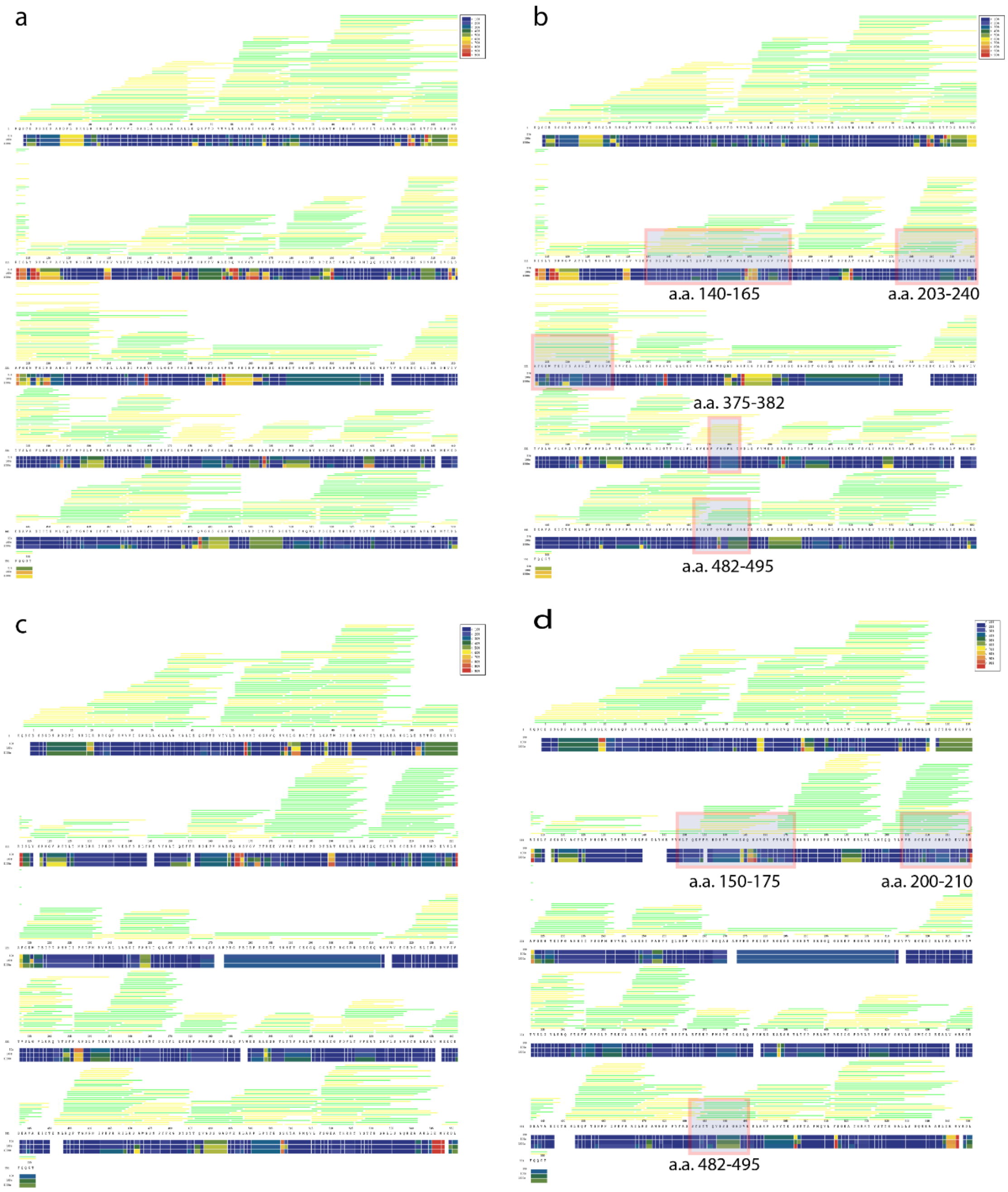

Supplementary Fig. 2. Sequence coverage and H/D exchange heat map of relative deuterium incorporation. Level of deuteration for hSMOX Apo (a, c), in complex with MDL72527 (b) and JNJ-1289 (d). Thin lines indicate sequence coverage on top of the sequence. Green indicates

confident peaks, yellow: medium-confident peaks (confidence based on theoretical isotope distribution of the chromatographic peak). The coverage for peptides used in HDX-MS analysis is 99.6% of the entire hSMOX. Under the protein sequence are heat maps in which each row represents the partially deuterated time points (10, 100 and 1000 sec). The color corresponds to the deuterated percentage of that area of protein per key on top right corner. Parts of the protein showing significant differences in deuterium uptake are highlighted in red boxes.

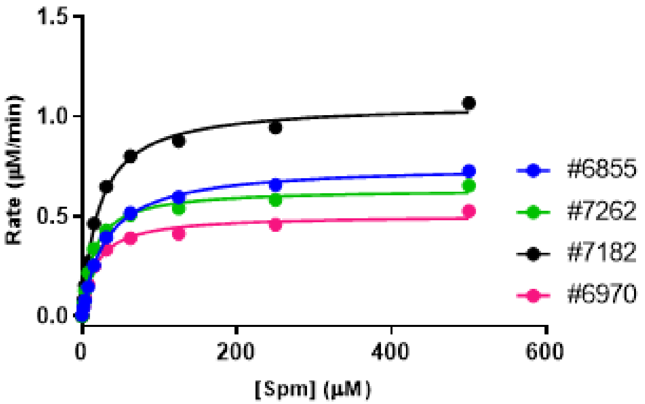

Enzyme	V_{max} (μM/min)	Spm K_m (μM)	k_{cat} $(s)^{-1}$	k_{cat}/K_m $(M^{-1}s^{-1})$
#6855	0.76	30.63	5.1	1.65E+05
#7262	0.64	15.26	4.3	2.80E+05
#7182	1.07	21.55	7.1	3.31E+05
#6970	0.51	17.41	4.8	2.77E+05

Supplementary Figure 3. Kinetic parameters of different engineered hSMOX constructs.

Steady-state kinetics of Spm for the different constructs employed in the HyPerBlu luminescence assay. The Spm concentrations were tested in the range of 0-500 μM using 2.5 nM SMOX for constructs #6855, #7262, and #7181 or 1.76 nM for construct #6970. These results showed that k_{cat}/K_m values of all the constructs were within two-fold of one another.

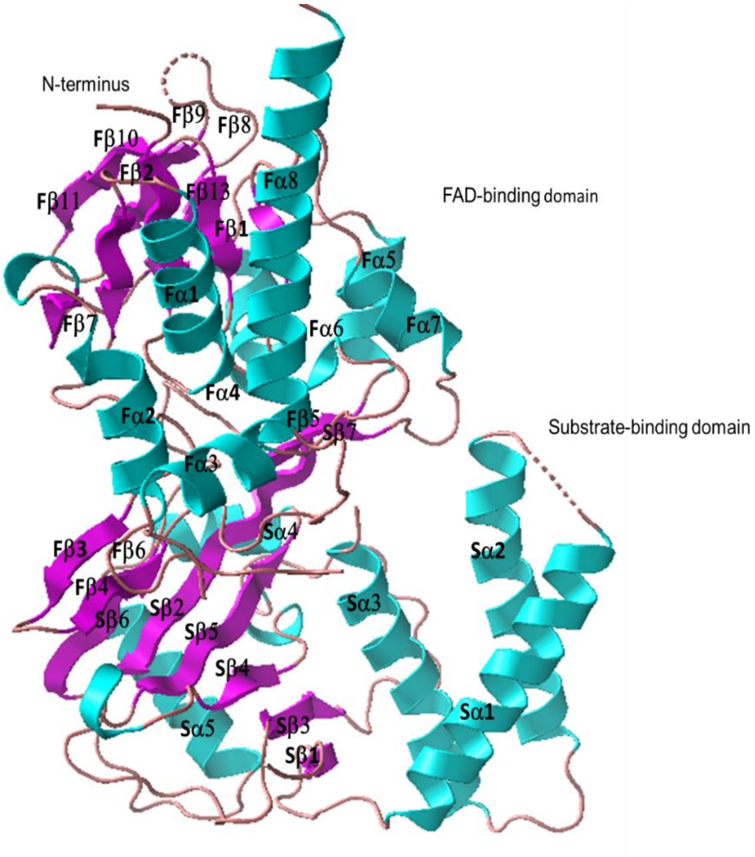

Supplementary Fig. 4. Crystal structure of ehSMOX. ehSMOXe consists of two domains (FAD-containing domain, F-domain; substrate-containing, S-domain). A–helices are depicted in cyan and β-sheets in magenta color for each domain.

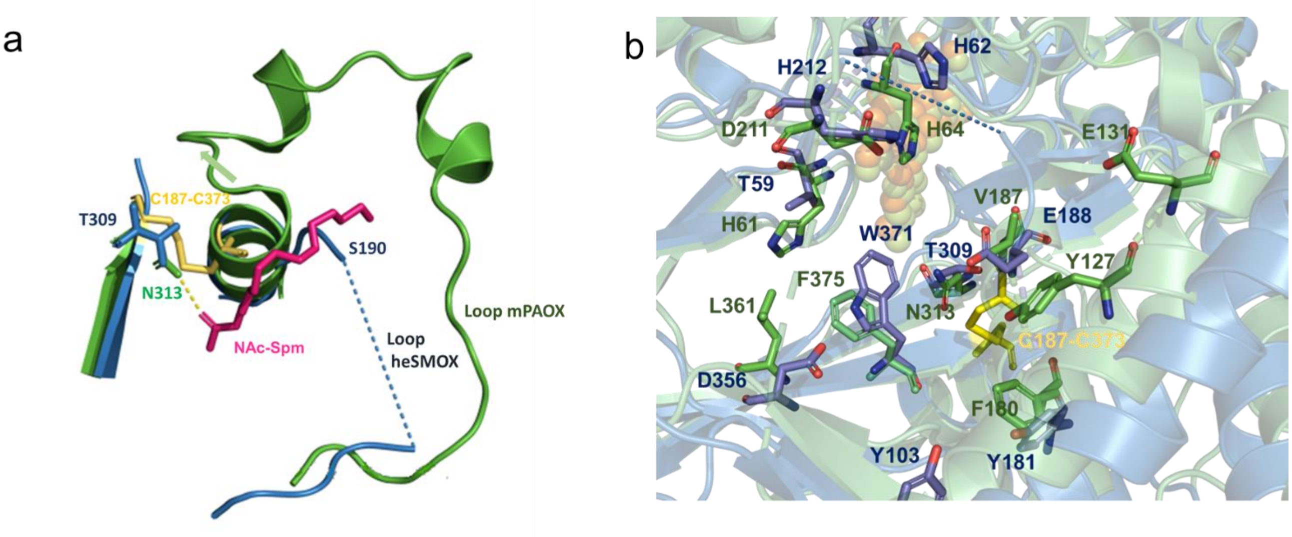

Supplementary Fig. 5. ehSMOX and mPAOX catalytic site. Overlay of ehSMOX, mPAOX in blue and green ribbon, respectively. **a,** S190 at the end of heSMOX Sα3 display orientation of the substrate-binding pocket loop different from that of mPAOX. Indeed, the heSMOX loop position clashes with N-AcSpm superimposed from mPAOX structure**. b,** Residues in the binding pocket different in ehSMOX and mPAOX are depicted in sticks (blue for ehSMOX and in green for mPAOX). FADs are in orange and green spheres for ehSMOX and mPAOX, respectively.

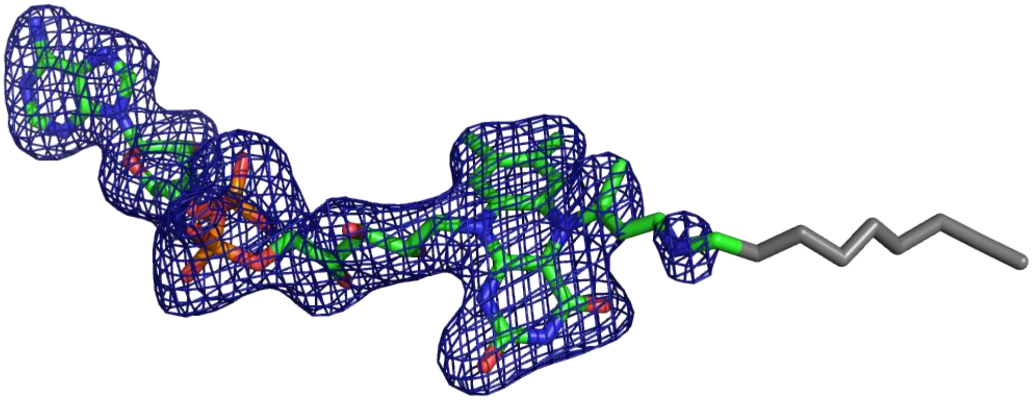

Supplementary Fig. 6. Electron Density for FAD-MDL72527. The final refined model for the FAD-MDL72527 covalent adduct is shown as stick figure. Atoms modelled with zero occupancy are shown in grey. Fo-Fc omit map is shown contoured at 2.5σ.

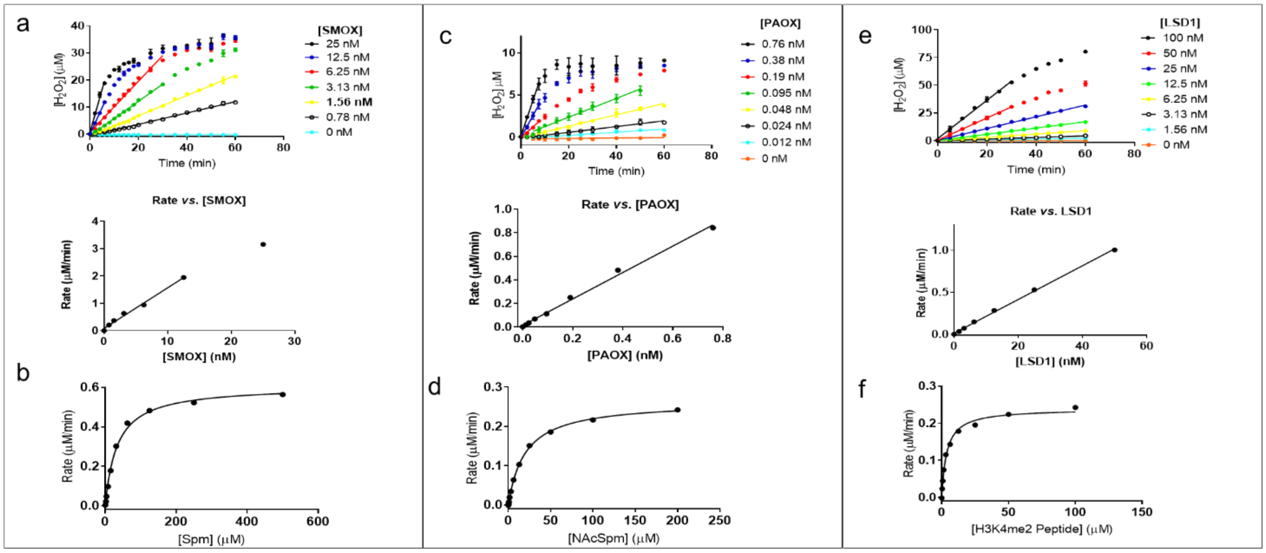

Supplementary Fig. 7. Characterization of enzyme activity and kinetics. **a,** hSMOX activity was measured in the HyPerBlu luminescence assay using 30 µM Spm at a range of enzyme concentrations (0 – 25 nM). Replots of rate profiles demonstrated linearity to 12.5 nM enzyme with an observed specific enzyme activity of 190 min^{-1}. **b**, Steady-state kinetics of Spm oxidation were determined at 2.5 nM hSMOX. The Spm concentrations were tested in the range of 0 - 500 µM. The resulting rate vs. [Spm] data were fit with the Michaelis-Menten equation and K_m and k_{cat} values were determined to be 33.9 ± 2.7 µM and 4 s^{-1}, respectively. **c,** hPAOX enzyme activity was measured in the HyPerBlu luminescence assay using 10 µM *N*-AcSpm at a range of enzyme concentrations (0 - 0.76 nM). Replots of rate profiles demonstrated linearity to 0.76 nM with an observed specific enzyme activity of 1350 min^{-1}. **d,** Steady-state kinetics of *N*-AcSpm oxidation was determined at 0.076 nM hPAOX. The *N*-AcSpm concentrations were tested in the range of 0 - 200 µM and the resulting data fit and analyzed as described above. The corresponding K_m and k_{cat} values were determined to be 20.1 ± 0.63 µM and 57 s^{-1}, respectively. **e,** LSD1 enzyme activity was measured in the HyPerBlu luminescence assay using 50 µM peptide substrate representing residues 1-21 of histone H3 containing a dimethylated lysine at position 4 (H3K4me2) at a range of enzyme concentrations (0 - 100 nM). Replot of LSD1 rate profile demonstrated linearity to 50 nM with an observed specific enzyme activity of 20 min^{-1}. **f,** Steady-state kinetics of H3K4me2 peptide oxidation was determined at 20 nM LSD1. The H3K4me2 peptide concentrations were tested in a range from 0 - 100 µM and the resulting data fit and analyzed as described above. The corresponding K_m and k_{cat} values were determined to be 3.8 ± 0.35 µM and 0.2 s^{-1}, respectively.

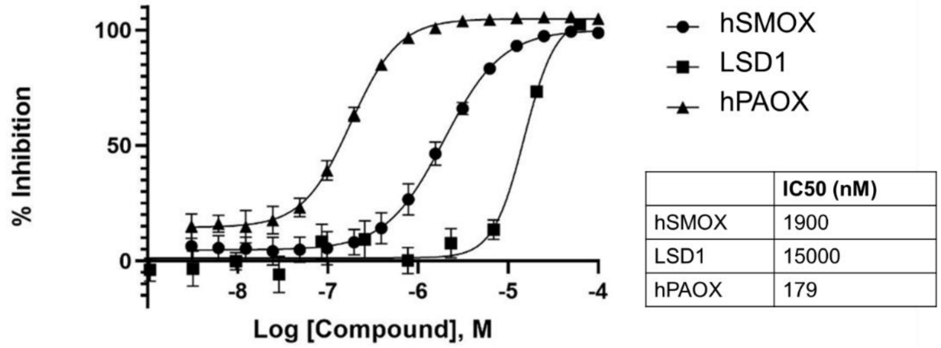

	IC50 (nM)
hSMOX	1900
LSD1	15000
hPAOX	179

Supplementary Fig. 8. Chlorhexidine specificity. Dose-response titrations of chlorhexidine. Inhibition of hSMOX, hPAOX and LSD1 with chlorhexidine was measured at 2.5 nM, 60 pM or 10 nM enzyme, respectively. Reactions were initiated with 30 µM Spm, 20 µM *N*-AcSpm or 8 µM histone H3K4me2 peptide and incubated for 1h for the reactions containing hSMOX, hPAOX and LSD1, respectively. JNJ-1289 inhibits hSMOX (black circles), hPAOX (black triangles), and LSD1 (black squares) with an IC_{50} of 1.9 µM, 0.179 µM and 15 µM, respectively.

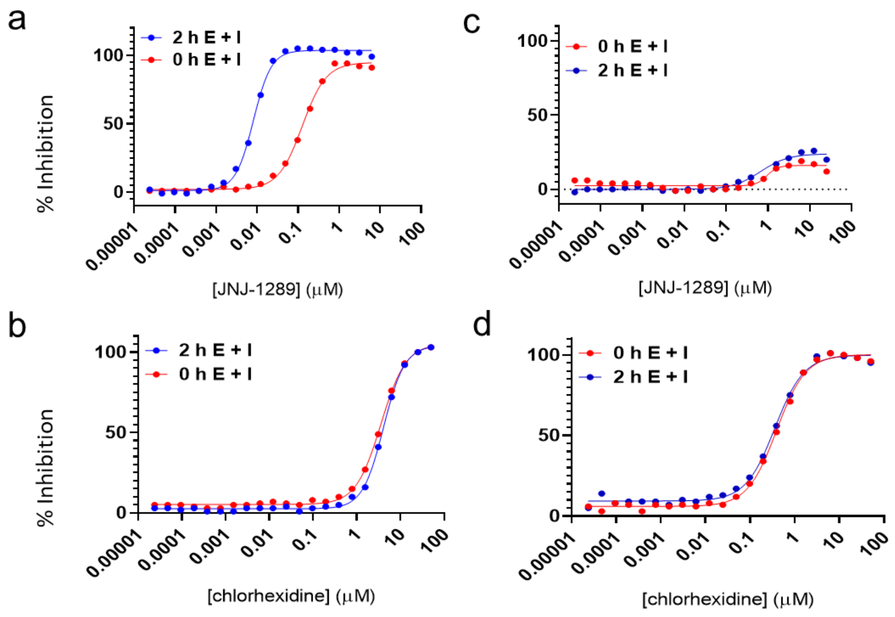

Inhibitor	hSMOX 0 h E + I IC_{50} (µM)	hSMOX 2 h E + I IC_{50} (µM)	hPAOX 0 h E + I IC_{50} (µM)	hPAOX 2 h E + I IC_{50} (µM)
JNJ-1289	0.13	0.01	≥ 50	≥ 50
chlorhexidine	3.6	4.1	0.39	0.36

Supplementary Fig. 9. Dose-response titrations of JNJ-1289 and chlorhexidine. JNJ-1289 and chlorhexidine ± 2h pre-incubation with the enzyme. Inhibition of hSMOX with **a,** JNJ-1289 and **b,** chlorhexidine was measured at 2.5 nM enzyme. Reactions were initiated with 30 µM Spm

and run for 1h. Inhibition of hPAOX with **c**, JNJ-1289 and **d,** chlorhexidine was measured with 60 pM enzyme. Reactions were initiated with 20 µM N-AcSpm and run for 1h. JNJ-1289 displayed selective and time-dependent inhibition of hSMOX. Chlorhexidine inhibited hSMOX and hPAOx and did not display time-dependent inhibition of either enzyme.

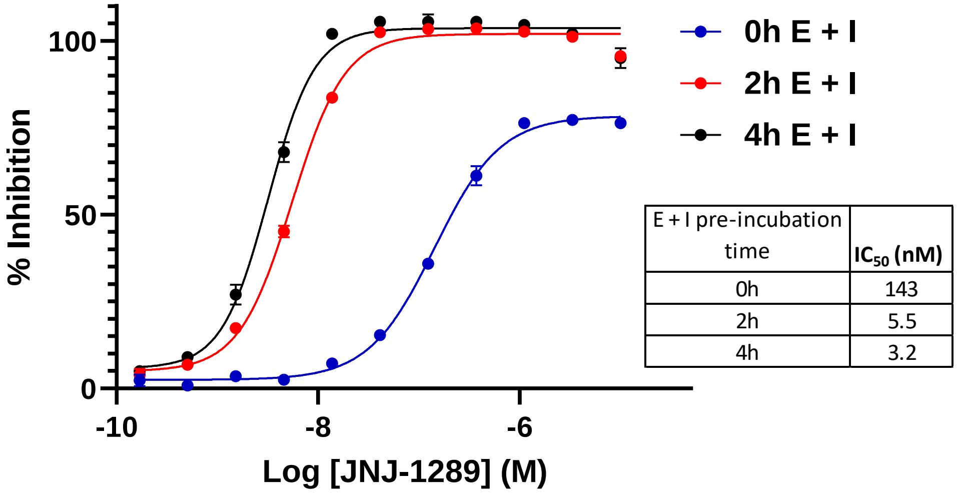

E + I pre-incubation time	IC_{50} (nM)
0h	143
2h	5.5
4h	3.2

Supplementary Fig. 10. Equilibrium IC_{50} of JNJ-1289. Dose-response titrations of JNJ-1289 for inhibition of hSMOX was measured using a 0h, 2h or 4h preincubation. Reactions were initiated with 30 µM Spm and incubated for 1h. The observed IC_{50} of 143 nM, 5.5 nM and 3.2 nM at 0h, 2h and 4h preincubation suggested that JNJ-1289 inhibited SMOX in a time-dependent manner. The small change in potency at 2h versus 4h suggested that compound is at equilibrium with hSMOx after 2h.

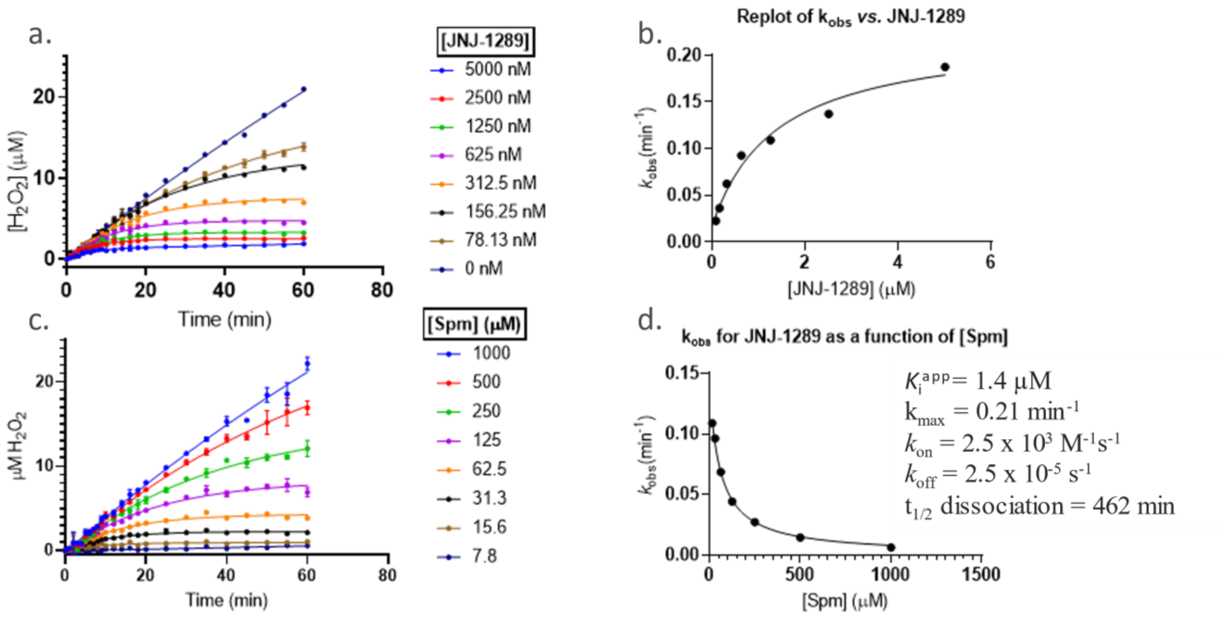

Supplementary Fig. 11. Kinetics of JNJ-1289. **a,** Reaction progress curve analysis of 2 nM hSMOX and 60 μM Spm obtained with a range of JNJ-1289 concentrations (0 - 5000 nM). Progress curves were monitored for 1h and fit to Eqn S1 at each JNJ-1289 concentration to determine the observed rate constant, k_{obs}.

$$[P] = Y0 + \upsilon_s t + \frac{\upsilon_i - \upsilon_s}{k_{obs}}(1 - e^{-k_{obs}t}) \qquad (S1)$$

where υ_i and υ_s are the initial and steady state velocities, respectively, t is time and k_{obs} represents the observed rate constant.

b, Replot of k_{obs} *vs*. [JNJ-1289] was hyperbolic, which indicated a two-step binding mechanism and fit to Eqn S2.

$$k_{obs} = \frac{k_5[I]}{K_{iapp} + [I]} + k_6 \qquad (S2)$$

where I is the inhibitor concentration, k_5 and $k6$ are the estimated forward and reverse rate constants, respectively, and K_{iapp} is the apparent value of the initial E·I complex.

c, Reaction progress curve analysis of 2 nM hSMOX and 312 nM JNJ-1289 with a range of Spm concentrations (7.8 – 1000 μM). Progress curves were monitored for 1h and fit to Eqn S3 at each Spm concentration to determine the mode of inhibition.

$$k_{obs} = \frac{k}{1+[S]/K_m} \qquad (S3)$$

where S is Spm concentration, K_m is the Michaelis-Menten constant and k is rate constant in the presence of JNJ-1289.

d, Replot of k_{obs} as a function of [Spm] showed a hyperbolic decrease, which indicated that JNJ-1289 is a competitive inhibitor.

a

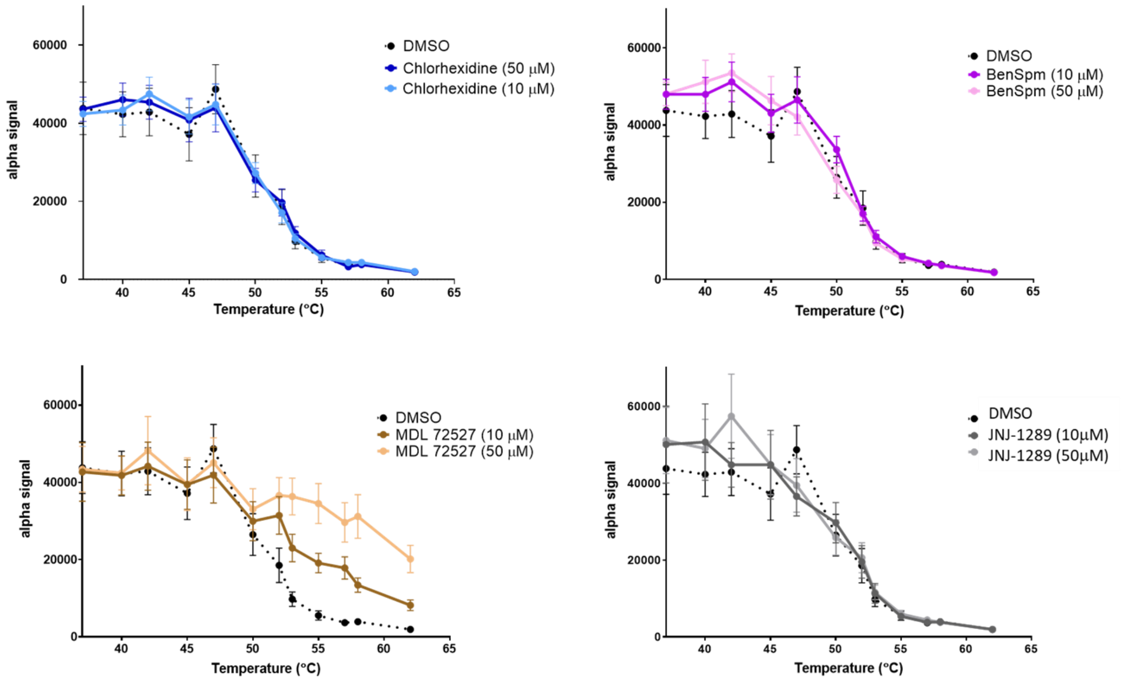

b

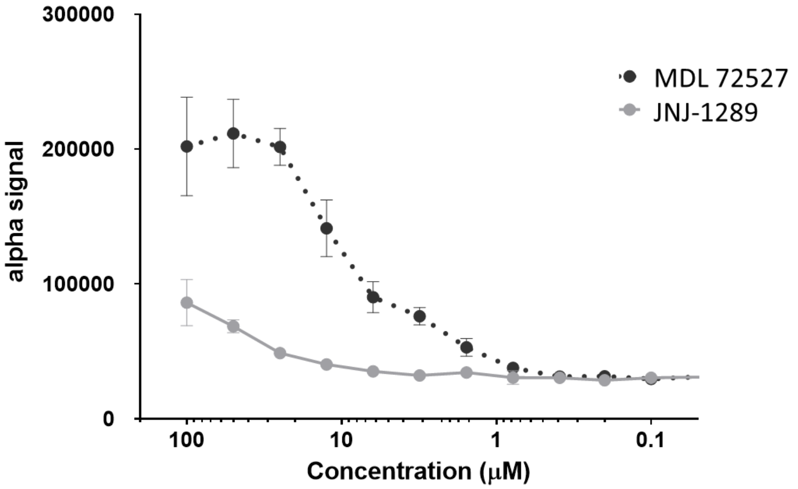

Supplementary Fig. 12. CETSA. Melting and shift curve analysis of hSMOX in A549 living cells treated with the three different compounds in saline solution. **a,** AlphaLISA signals from A549 living cells treated with chlorhexidine, BenSpm, MDL 72527 and JNJ-1289 at two concentrations, 10 and 50 µM. Samples were heated to temperatures ranging from 37°C to 63°C. As a control, 1% DMSO was used (dotted line). CETSA has been executed with 3 individual

experiments and 4 technical replicates per experiment. **b**, AlphaLISA signals from A549 living cells treated with MDL 72527 and JNJ-1289 in a concentration range of 100 µM- 0.05 µM and heated to 54 °C. (1 individual experiment and 4 technical replicates).

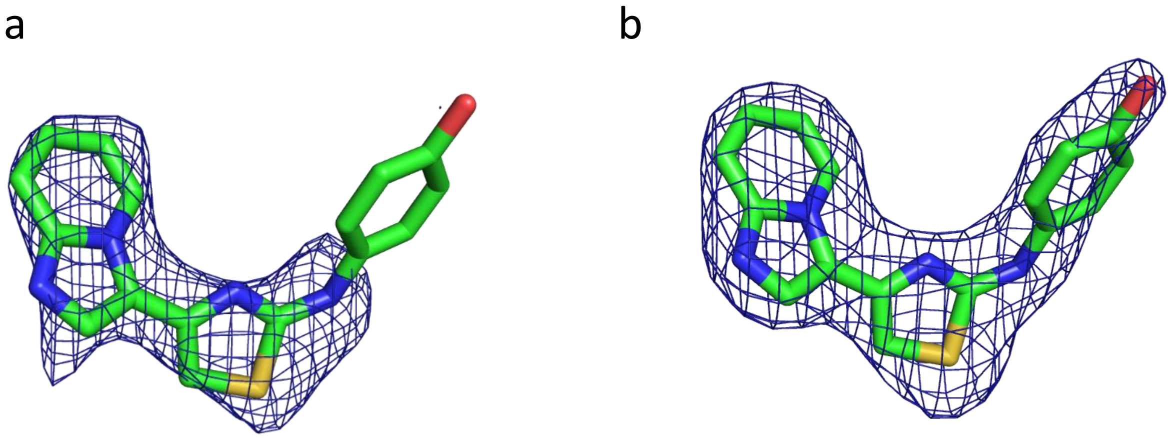

Supplementary Fig. 13. Electron Density for JNJ-1289. The final refined model for JNJ-1289 is shown as stick figure. **a,** Fo-Fc omit map contoured at 2.5σ. **b,** 2Fo-Fc map contoured at 1.0σ.

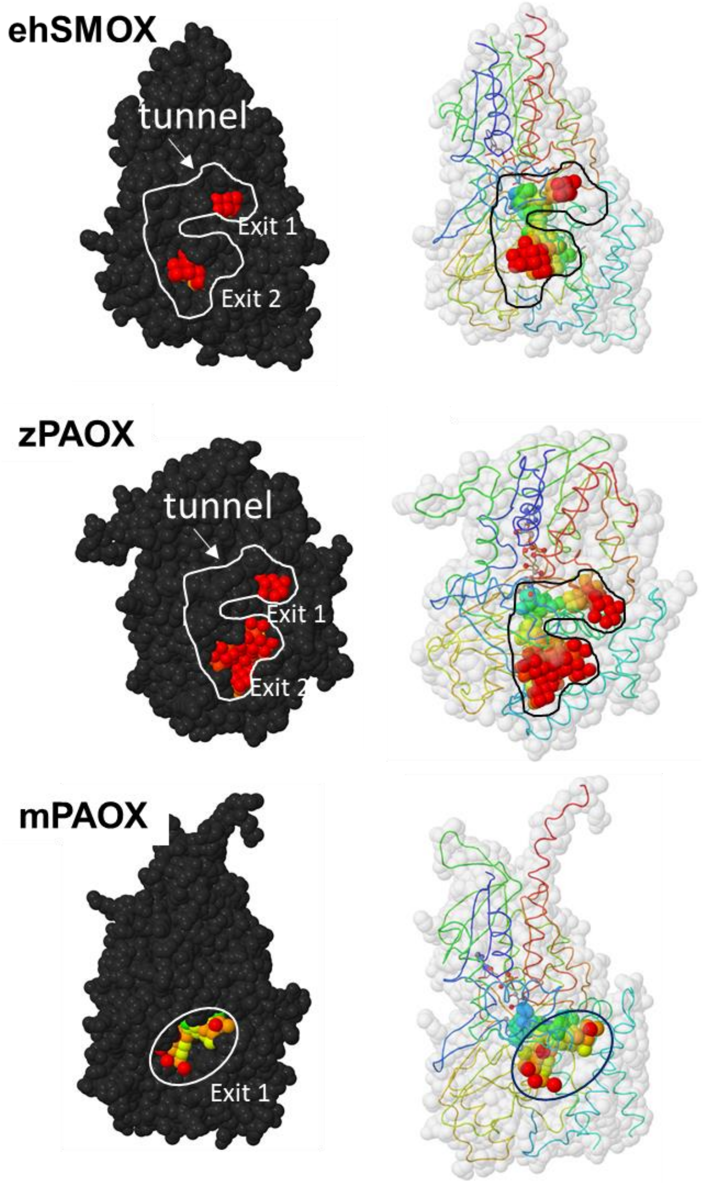

Supplementary Fig. 14. Representation of the substrate cavities for ehSMOX, mPAOX (PDB ID: 5LAE) and zPAOX (PDB ID: 1B37). Active site cavities representation has been obtained using *Pockets And Cavities Using Pseudoatoms in Proteins* (PACUPP) a free, open-source program for identifying and visualizing cavities in macromolecules. Pseudoatoms are depicted as spheres and color-coded by depth from the surface with blue (deepest) to red (surface exposed), while in dark gray are the atom positions (on the left). On the right, the protein surface is depicted in light gray and superimposed with its cartoon-loop representation of the protein and pseudoatoms represented as in the correspondent right figure. ehSMOX loop position in the bound form creates a cavity shape more similar in conformation (U shape depicted as a tunnel with the two exits) and depth to zPAOX than to mPAOX.

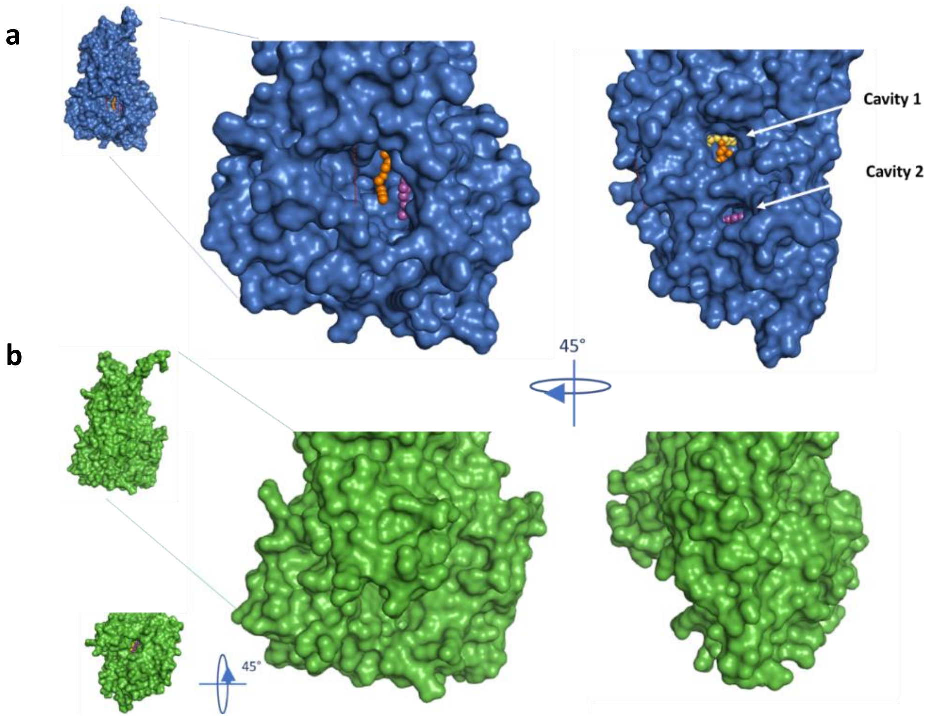

Supplementary Fig. 15 Cavities in heSMOX and mPAOX. a, surface representation of heSMOX bound to JNJ-1289 in blue. FAD, MDL and JNJ-1289 are depicted in yellow, orange and magenta spheres, respectively, while N-AcSpm is in light green spheres. Red dashed line represents the missing loop aa 81-97._**b**, surface representation of mPAOX (PDB: 5MBX) in green, FAD and N-AcSpm are depicted in yellow and purple spheres, respectively.

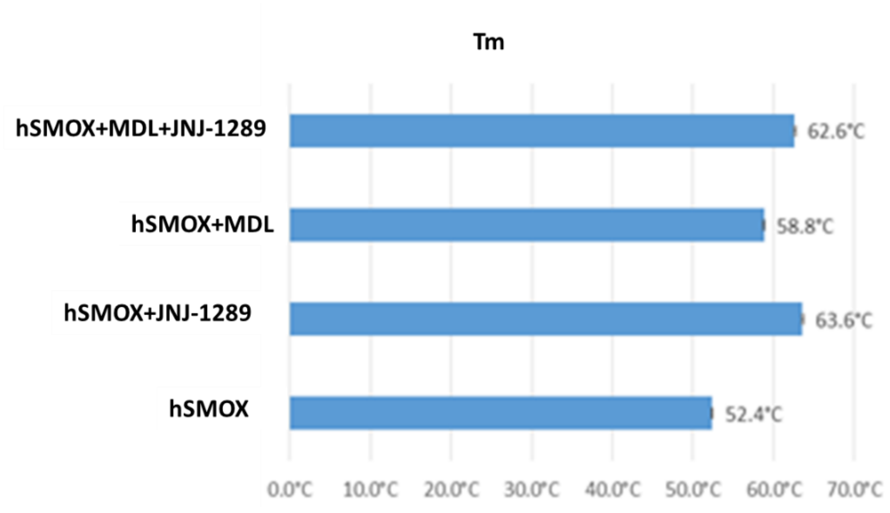

Supplementary Fig. 16. **NanoDSF experiments**. Results for SMOX in presence of 10-fold molar excess of MDL72527 and/or JNJ-1289. Tm for SMOX in each experiment is noted at the end of the bar.

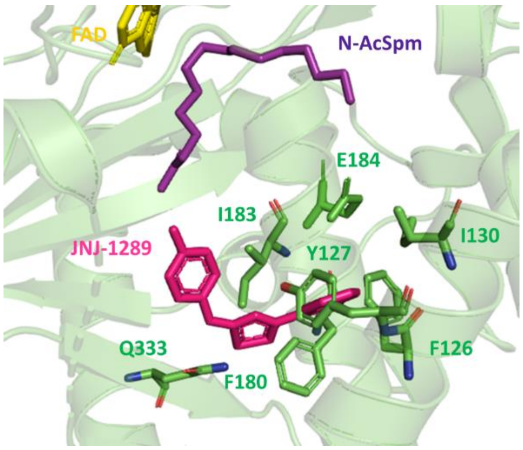

Supplementary Fig.17. JNJ-1289 allosteric binding pocket. Overlay of JNJ-1289 with mPAOX (PDB: 5MBX). Residues on mPAOX near JNJ-1289 are depicted in green stick. JNJ-1289 is indicated in hot pink, N-AcSpm in purple and FAD in yellow sticks, respectively. Y127 in mPAOX would not allow for the binding of JNJ-1289.

a

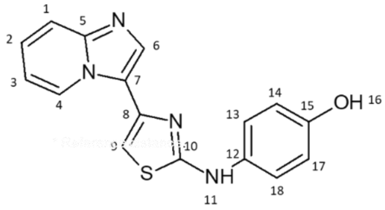

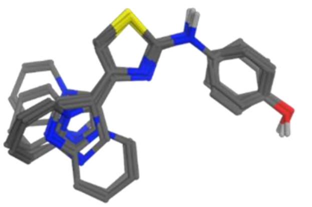

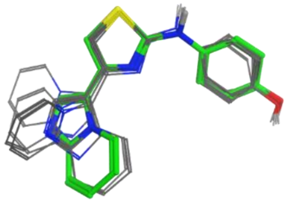

Atom	^{13}C Chemical Shift (δ ppm)	^{1}H Chemical Shift (δ ppm); multiplicity, *J* Coupling (Hz)
1	112.7	8.02; dt, *J* = 9.1, 1.2 Hz
2	132.3	7.97; ddd, *J* = 9.1, 6.8, 1.2 Hz
3	116.6	7.58; ddd, *J* = 6.9, 6.8, 1.2 Hz
4	127.8	9.44; dt, *J* = 6.9, 1.2, 1.2 Hz
5	139.8	-
6	121.1	8.59; s
7	121.9	-
8	136.8	-
9	106.1	7.46; s
10	165.2	
11	-	10.19
12	132.4	-
13,18	119.6	7.40; m
14,17	115.1	6.78; m
15	152.8	
16	-	9.22; s (br)

Atom 1	Atom 2	Normalized integral	Corrected integral	Calculated distance (Å)
4	3	31.6	31.6	2.46*
4	11	0.7	0.7	4.64
4	9	13.0	13.0	2.85
4	13,18	12.0	6.0	3.24
11	9	0.2	0.2	5.59
11	13,18	29.0	14.5	2.80
6	9	13.8	13.8	2.82
6	13,18	1.0	0.5	4.91
6	11	No NOE		

b

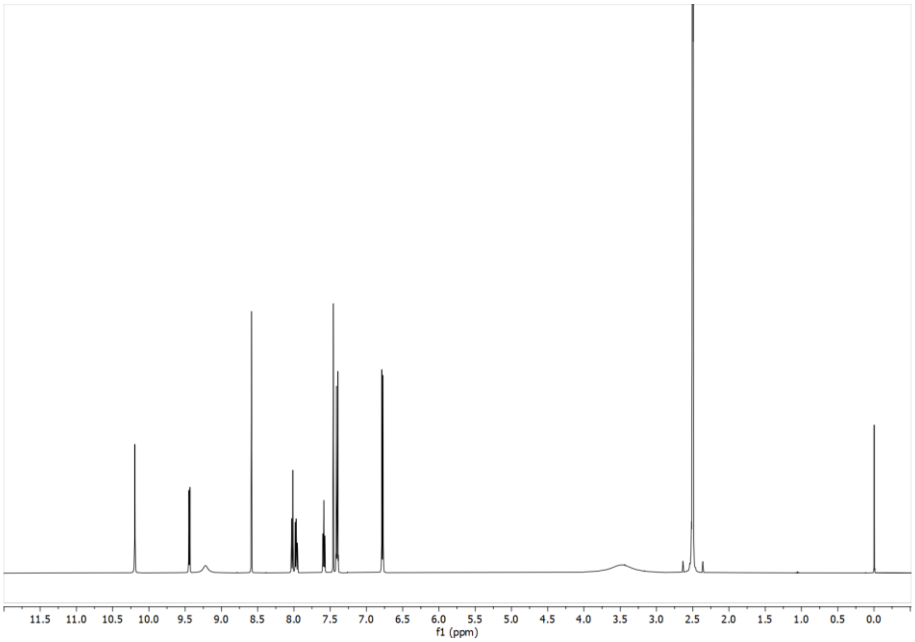

c

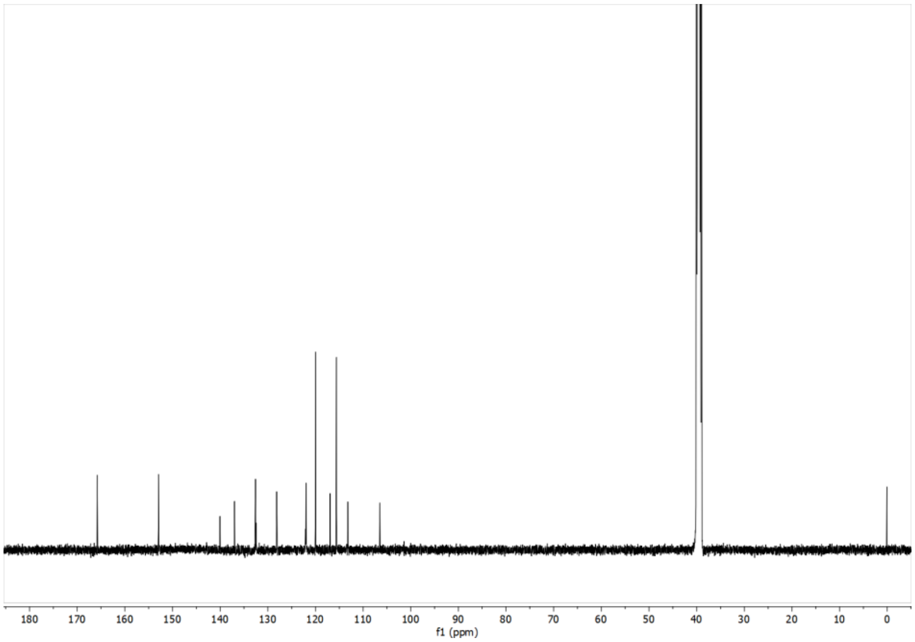

Supplementary Fig.18. Nuclear Magnetic Resonance solution conformation of JNJ-1298.

a, Structure of JNJ-1289 and in the upper right the overlay of the X-ray pose of JNJ-1289 (green) with the solution conformers (gray). JNJ-1289 NMR assignments in DMSO-d_6 at 298 K, NOE correlations and inter-proton distances (Å) calculated from 2D EASY-ROESY (DMSO-d_6).
b, JNJ-1289 ^{1}H NMR (500 MHz, DMSO-d_6): δ 10.18 (s, ^{1}H), 9.43 (m, ^{1}H), 9.22 (s, 1H), 8.56 (s, ^{1}H), 8.01 (dt, J = 9.1, 1.2 Hz, ^{1}H), 7.95 (ddd, J = 9.1, 6.9, 1.2 Hz, ^{1}H), 7.57 (td, J = 6.9, 1.2 Hz, ^{1}H), 7.45 (s, 1H), 7.40 (m, 2H), 6.78 (m, 2H); **c,** JNJ-1289 ^{13}C NMR (125 MHz, DMSO-d_6): d 165.8, 152.9, 140.0, 137.0, 132.6, 132.5, 128.2, 122.1, 122.0, 120.0, 116.9, 115.6, 113.2, 106.5.

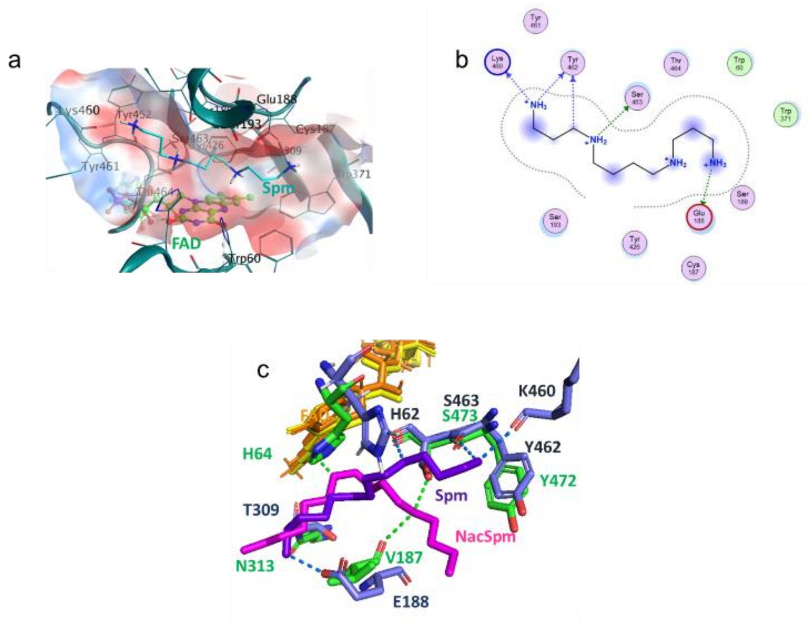

Supplementary Fig.19. Binding model of spermine in ehSMOX catalytic site.

a, Substrate binding pocket with electrostatically negative charged surface colored red and positive in blu, Spm depicted in cyan sticks and the FADin green spheres. **b**, Spm hydrogen bonding network. **c,** heSMOX residues (in blue sticks) forming H-bond with modeled Spm (in purple) superimposed with mPAOX residues (in green) forming H-bonds with N-AcSpm (in magenta). FAD in heSMOX and mPAOX is represented in orange and yellow sticks, respectively.

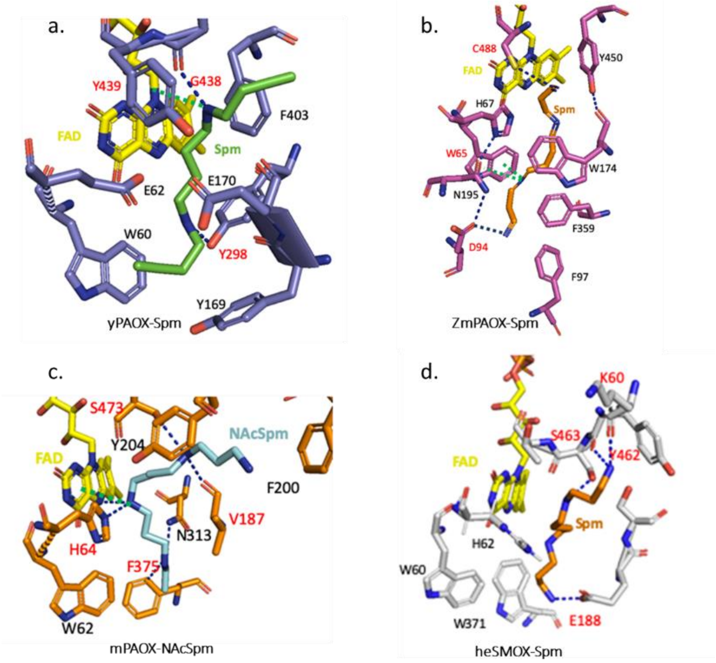

Supplementary Fig. 20. Comparison of substrate binding in polyamine oxidases.

a. *Saccharomyces cerevisiae* polyamine oxidase (yPAOX) in complex with Spm in green sticks (PDB ID:1XPQ); **b**. Zea mays polyamine oxidase (ZmPAOX) in complex with Spm in orange sticks (PDB ID:1B5Q); **c.** mPAOX in complex with N-Ac-Spm in cyan sticks (PDB ID:5MBX). **d**. Spm bound model of heSMOX.

FAD is in yellow sticks, and in red are labeled residues involved in H-boding or pi-cation interactions with the correspondent substrate.

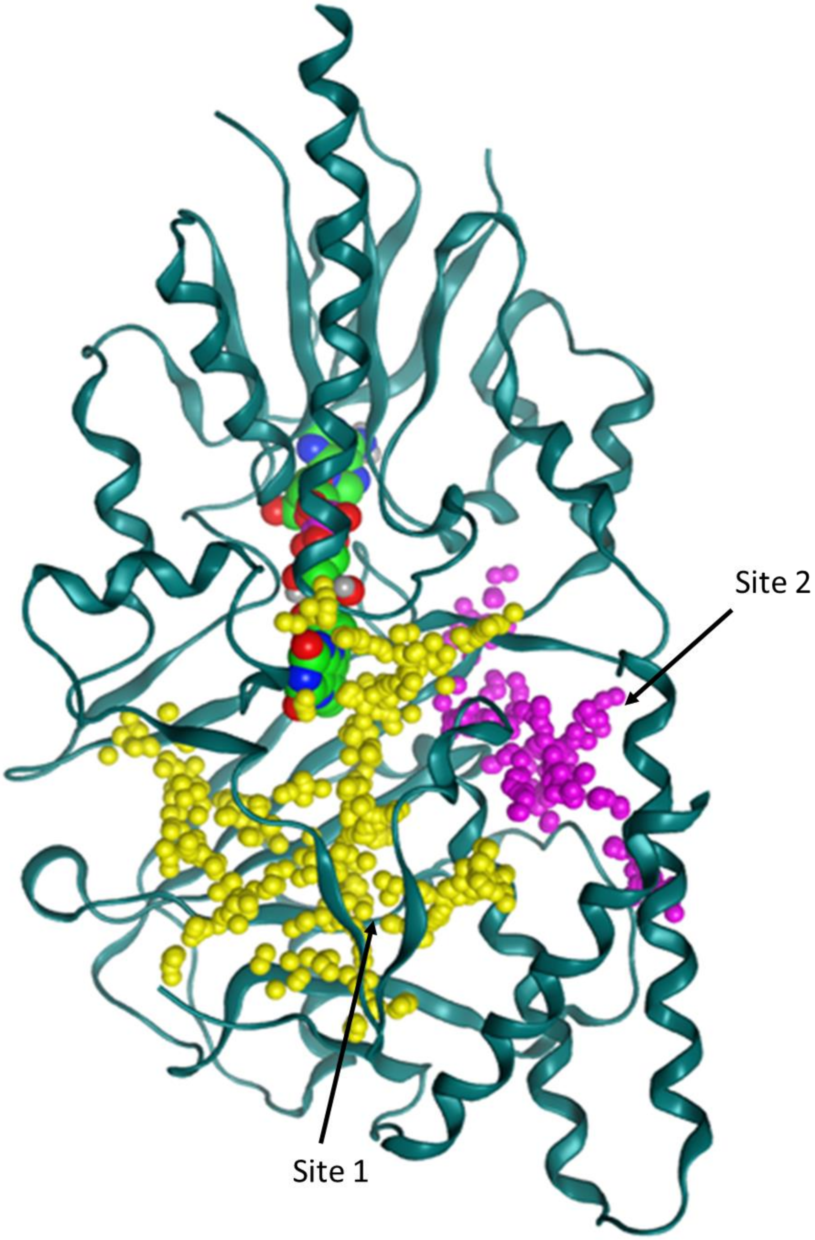

Supplementary Fig.21. ehSMOX druggable sites.

Druggable sites have been obtained with Sitefinder functionality in MOE. hSMOX is represented in teal green ribbon, FAD is indicated in green spheres, and the two most critical druggable sites are depicted with spheres yellow for SITE1 and magenta for SITE2 (pink).
