# Supplementary material for: Structure of human spermine oxidase in complex with a highly selective allosteric inhibitor
Source: Commun Biol. 2022 Aug 5;5:787. doi: 10.1038/s42003-022-03735-9 (PMC9355956; doi:10.1038/s42003-022-03735-9)
Supplement: Supplementary file 3 — Description of Additional Supplementary Files [file 42003_2022_3735_MOESM3_ESM.pdf]

Description of Additional Supplementary Files

File name: Supplementary Data
Description: High-resolution mass spectrometry data.
