# Supplementary material for: Structure of human spermine oxidase in complex with a highly selective allosteric inhibitor
Source: Commun Biol. 2022 Aug 5;5:787. doi: 10.1038/s42003-022-03735-9 (PMC9355956; doi:10.1038/s42003-022-03735-9)

Openlynx Report - jllaveri

Vnumber:jllaveri_858_1#1-1 Report:jllaveri Description:
Vial:4:9 Date:30-Jun-2022 Time:12:26:44
Instrument:XEVO-G2SQTOF#NotSet Method:C:\MassLynx\2BEH_TOF_P5 @S7010S7037.olp

Printed: Thu Jun 30 13:56:59 2022

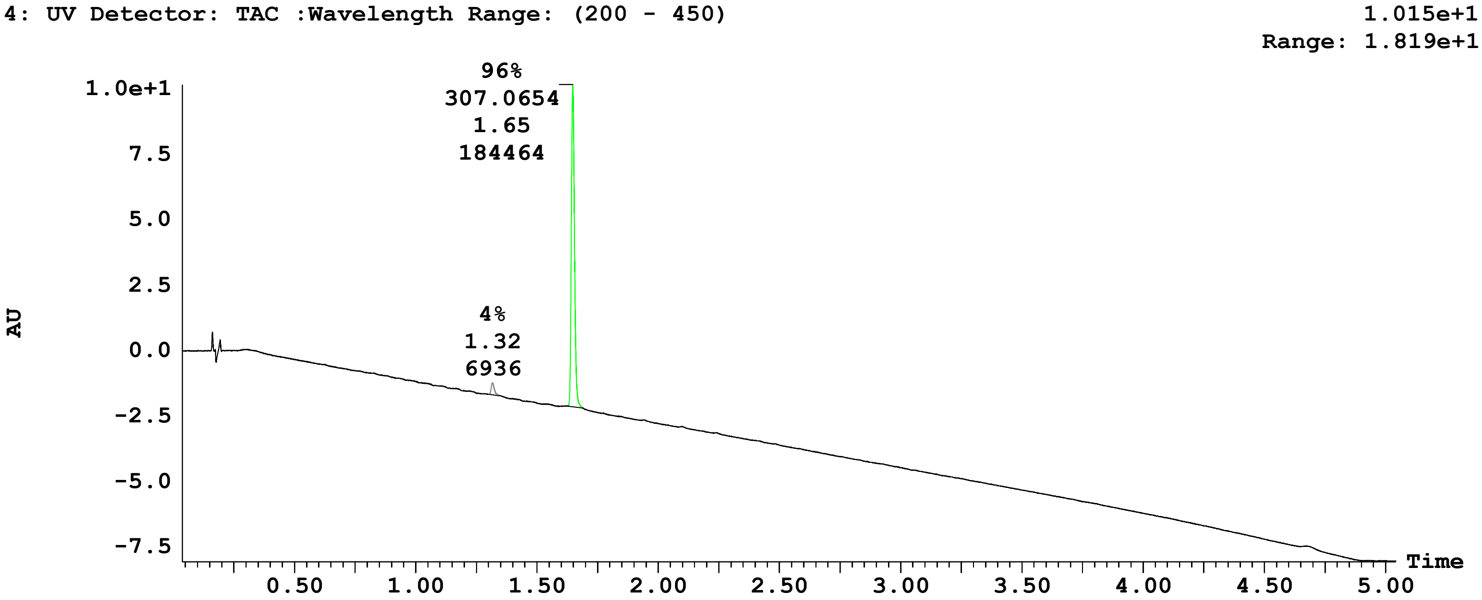

Peak Number	Time	State	Area %Total	Mass Found	AreaAbs
2	1.32		3.62		6936
3	1.65	OK	96.38	308.07, 308.07	184464

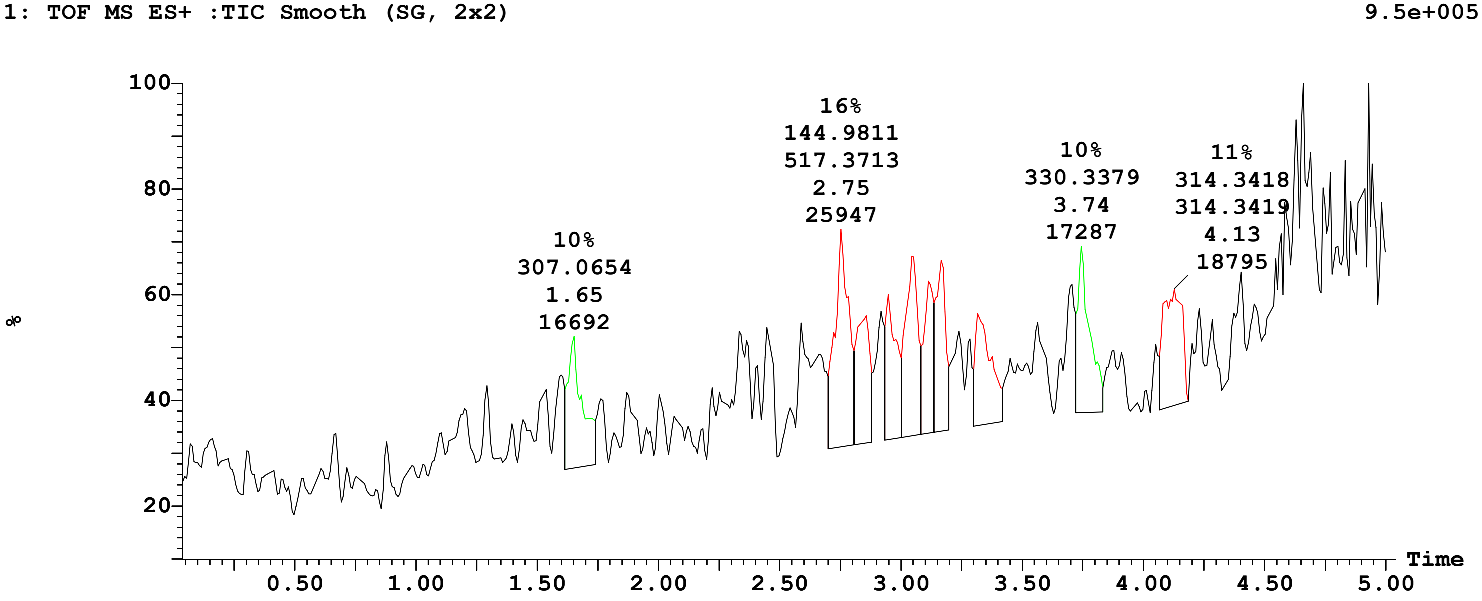

Vnumber:jllaveri_858_1#1-1 Report:jllaveri Description:
Vial:4:9 Date:30-Jun-2022 Time:12:26:44
Instrument:XEVO-G2SQTOF#NotSet Method:C:\MassLynx\2BEH_TOF_P5 @S7010S7037.olp

Printed: Thu Jun 30 13:56:59 2022

2: TOF MS ES+ :TIC Smooth (SG, 2x2) 8.4e+005

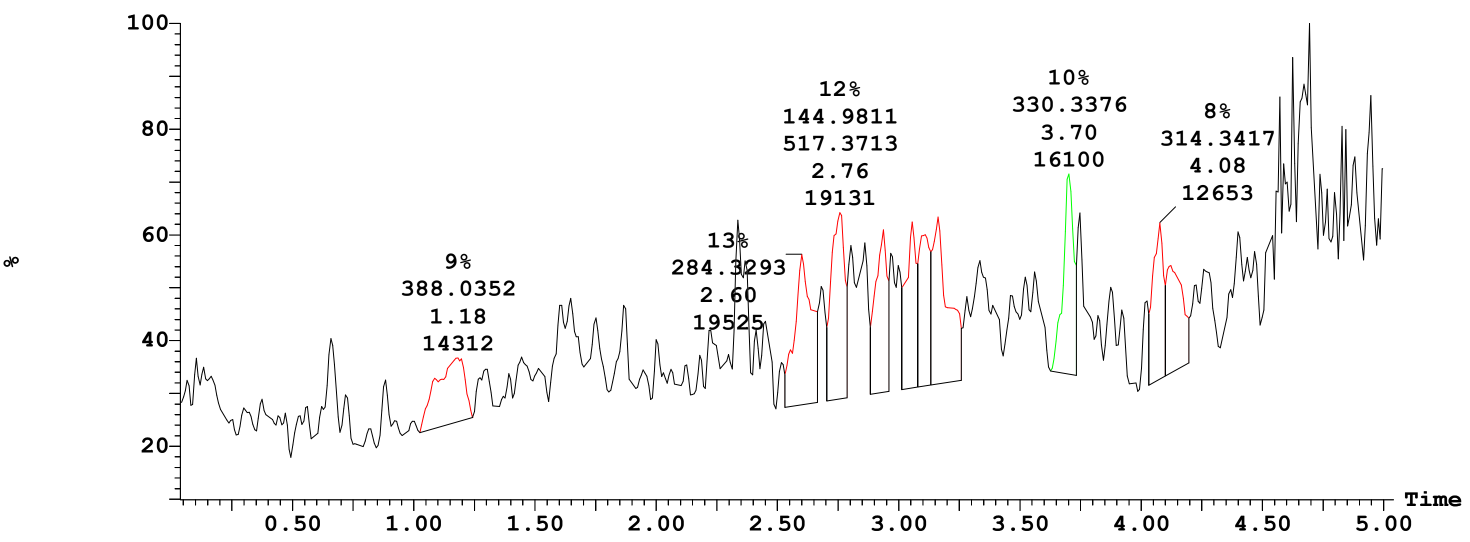

Peak ID	Time	Mass found	BPM	State
3	1.65	309.08,309.08	307.1	OK

3: (Time: 1.65) Combine (189:195) 1:TOF MS ES+ 4.2e+005

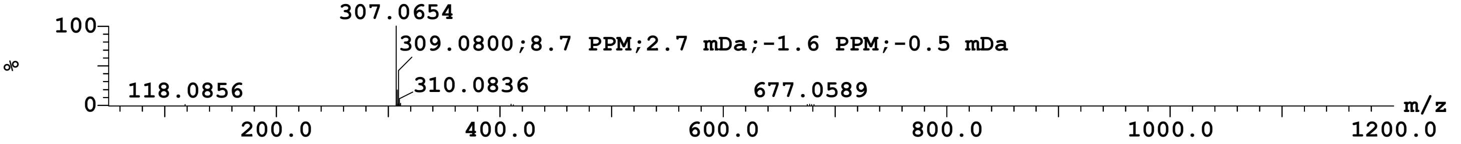

Peak ID	Time	Mass found	BPM	State
6	2.86	Not Found	258.3	Diversity fail

6: (Time: 2.86) Combine (331:335) 1:TOF MS ES+ 1.6e+004

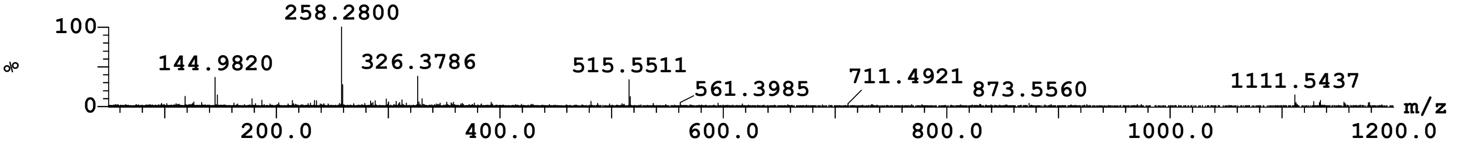

Peak ID	Time	Mass found	BPM	State
7	2.95	Not Found	326.4	Diversity fail

7: (Time: 2.95) Combine (341:347) 1:TOF MS ES+ 7.3e+004

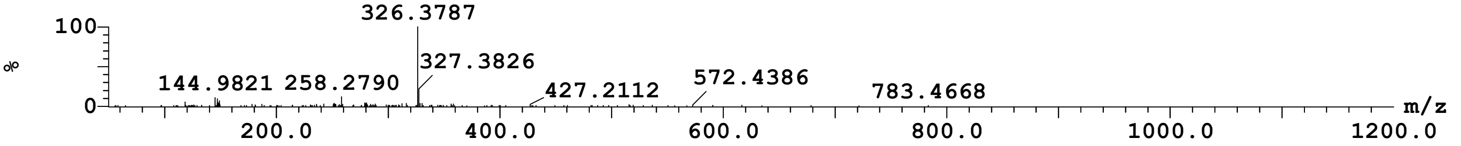

Vnumber:jllaveri_858_1#1-1	Report:jllaveri	Description:
Vial:4:9	Date:30-Jun-2022	Time:12:26:44
Instrument:XEVO-G2SQTOF#NotSet	Method:C:\MassLynx\2BEH_TOF_P5 @S7010S7037.olp	

Printed: Thu Jun 30 13:56:59 2022

Peak ID	Time	Mass found	BPM	State
8	3.04	Not Found	326.4	Diversity fail

8:(Time: 3.04) Combine (353:357) 1:TOF MS ES+ 3.0e+004

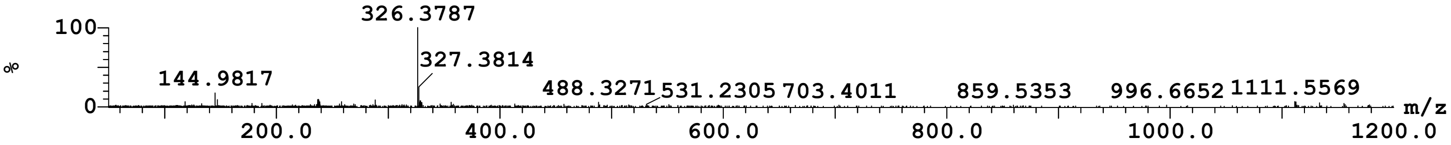

Peak ID	Time	Mass found	BPM	State
9	3.11	Not Found	326.4	Diversity fail

9:(Time: 3.11) Combine (360:366) 1:TOF MS ES+ 3.3e+004

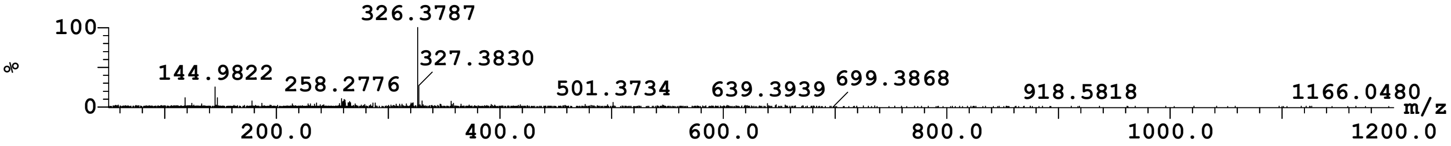

Peak ID	Time	Mass found	BPM	State
10	3.17	Not Found	326.4	Diversity fail

10:(Time: 3.17) Combine (367:373) 1:TOF MS ES+ 2.8e+004

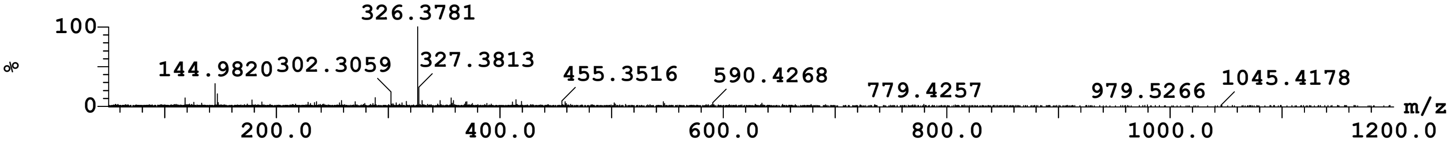

Peak ID	Time	Mass found	BPM	State
13	3.74	[illegible]9.08,331.06,350.10,309.08,331.06,350.11	330.3	OK

13:(Time: 3.74) Combine (435:440) 1:TOF MS ES+ 3.7e+004

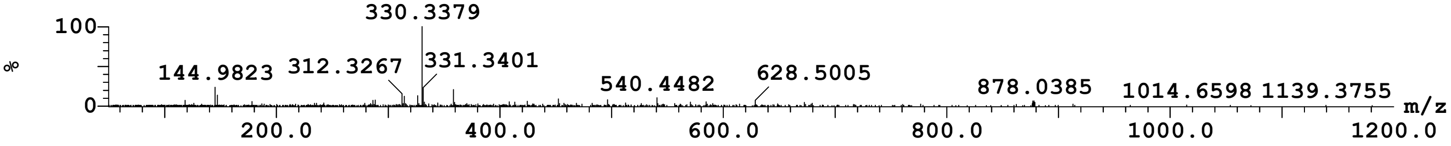

Peak ID	Time	Mass found	BPM	State
15	4.13	Not Found	314.3	Diversity fail

15:(Time: 4.13) Combine (480:484) 1:TOF MS ES+ 9.8e+003

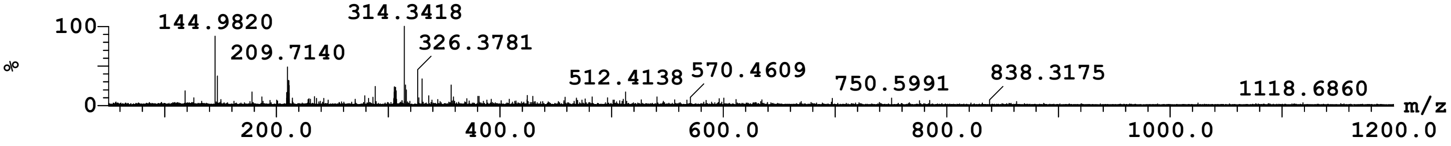

Vnumber:jllaveri_858_1#1-1 Report:jllaveri Description:
Vial:4:9 Date:30-Jun-2022 Time:12:26:44
Instrument:XEVO-G2SQTOF#NotSet Method:C:\MassLynx\2BEH_TOF_P5 @S7010S7037.olp

Printed: Thu Jun 30 13:56:59 2022

Peak ID	Time	Mass found	BPM	State
7	2.95	Not Found	326.4	Diversity fail

7:(Time: 2.94) Combine (339:345) 2:TOF MS ES+ 3.3e+004

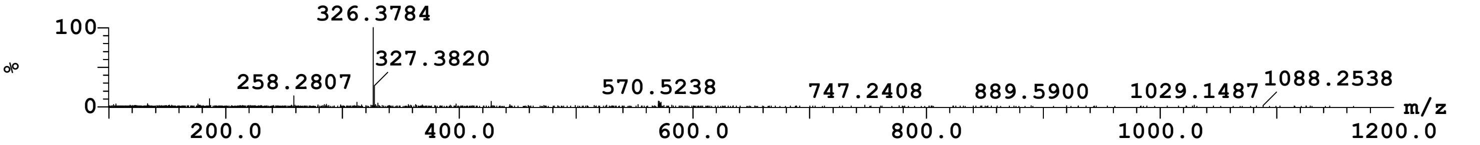

Peak ID	Time	Mass found	BPM	State
8	3.04	Not Found	326.4	Diversity fail

8:(Time: 3.06) Combine (353:358) 2:TOF MS ES+ 2.1e+004

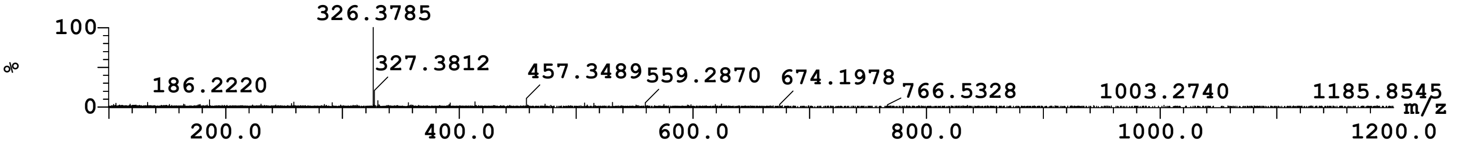

Peak ID	Time	Mass found	BPM	State
9	3.11	Not Found	326.4	Diversity fail

9:(Time: 3.11) Combine (359:365) 2:TOF MS ES+ 2.0e+004

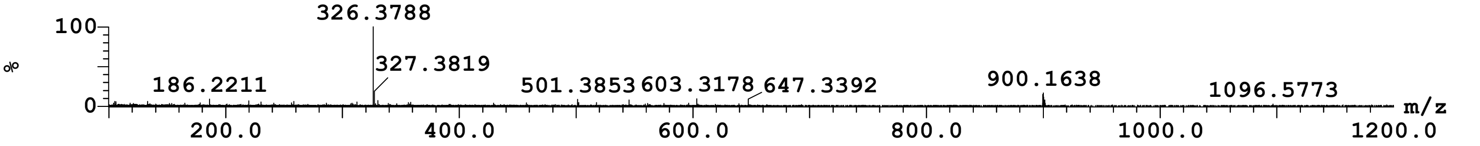

Peak ID	Time	Mass found	BPM	State
10	3.17	Not Found	326.4	Diversity fail

10:(Time: 3.16) Combine (366:372) 2:TOF MS ES+ 1.7e+004

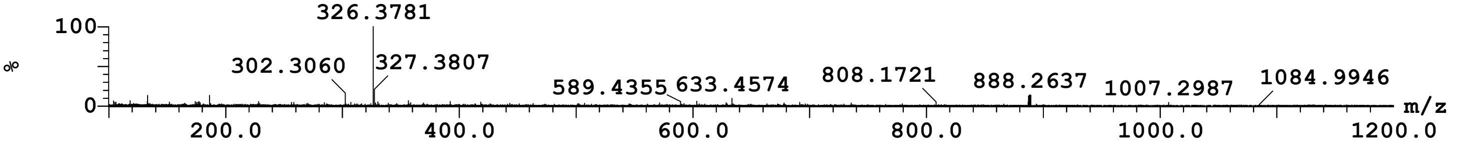

Peak ID	Time	Mass found	BPM	State
12	3.70	[illegible]0.10,617.15,309.08,331.06,350.11,617.15	330.3	OK

12:(Time: 3.70) Combine (429:435) 2:TOF MS ES+ 1.2e+004

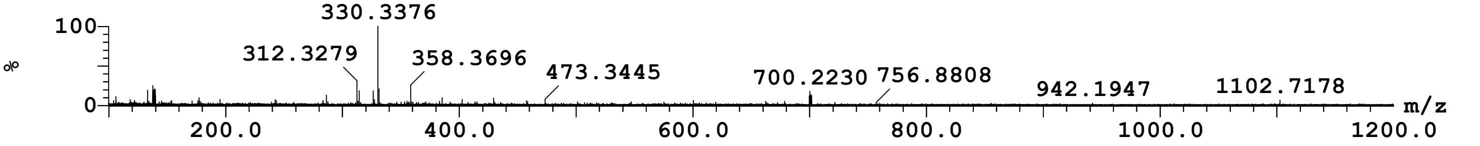

Supplement: Supplementary file 4 — Supplementary Data [file 42003_2022_3735_MOESM4_ESM.pdf]
